# Targeting the Neurokinin Receptor 1 with Aprepitant: A Novel Antipruritic Strategy

**DOI:** 10.1371/journal.pone.0010968

**Published:** 2010-06-04

**Authors:** Sonja Ständer, Dorothee Siepmann, Ilka Herrgott, Cord Sunderkötter, Thomas A. Luger

**Affiliations:** Department of Dermatology, Neurodermatology and Competence Center Pruritus, University of Münster, Münster, Germany; The University of Queensland, Australia

## Abstract

**Background:**

Chronic pruritus is a global clinical problem with a high impact on the quality of life and lack of specific therapies. It is an excruciating and frequent symptom of e.g. uncurable renal, liver and skin diseases which often does not respond to conventional treatment with e.g. antihistamines. Therefore antipruritic therapies which target physiological mechanisms of pruritus need to be developed. Substance P (SP) is a major mediator of pruritus. As it binds to the neurokinin receptor 1 (NKR1), we evaluated if the application of a NKR1 antagonist would significantly decrease chronic pruritus.

**Methods and Findings:**

Twenty hitherto untreatable patients with chronic pruritus (12 female, 8 male; mean age, 66.7 years) were treated with the NKR1 antagonist aprepitant 80 mg for one week. 16 of 20 patients (80%) experienced a considerable reduction of itch intensity, as assessed by the visual analog scale (VAS, range 0 to 10). Considering all patients, the mean value of pruritus intensity was significantly reduced from 8.4 VAS points (SD +/−1.7) before treatment to 4.9 VAS points (SD +/−3.2) (p<0.001, CI 1.913–5.187). Patients with dermatological diseases (e.g. atopic diathesis, prurigo nodularis) had the best profit from the treatment. Side-effects were mild (nausea, vertigo, and drowsiness) and only occurred in three patients.

**Conclusions:**

The high response rate in patients with therapy refractory pruritus suggests that the NKR1 antagonist aprepitant may indeed exhibit antipruritic effects and may present a novel, effective treatment strategy based on pathophysiology of chronic pruritus. The results are promising enough to warrant confirming the efficacy of NKR1 antagonists in a randomized, controlled clinical trial.

## Introduction

Chronic pruritus is a frequent and globally occurring symptom of systemic, dermatologic, neurological and psychiatric diseases [1]–[3]. It is currently estimated that 20 to 27% of all adults worldwide endure chronic pruritus [4]–[6]. Since the symptom is regularly characterized by a high intensity and long duration as well as by cutaneous self-injury due to scratching, it has a high impact on the quality of life and may lead to depression or even suicide of the sufferers [4], [7], [8]. Given that pruritus was regarded for a long time as subquality of pain [1], not much attention was paid to the neurobiological basis of the symptom in the past. A second reason for the lack of pursuit of specific treatment strategies was owed to the assumption that treatment of underlying disease would automatically relieve pruritus [2]. Therefore the mainstays of treatment for chronic pruritus until to date are still antihistamines, topical and systemic corticosteroids, or certain antidepressants [2]. However, their efficacy is limited and systemic application of corticosteroids and antidepressants may be associated with severe side-effects. Recent studies have provided evidence that pruritus has a different pathophysiology than pain and that it does not parallel the course of the underlying disease [1], [2]. Therefore we [9] and others [10] have pursued the concept to develop target-specific treatments targeting pathophysiological mechanism specific for pruritus. For example, in 2009, nalfurafine (which functions by activation of the spinal kappa-opioid-receptor) was licensed in Japan as the first oral drug against pruritus in hemodialysis patients [10]. Still, the development of new and targeted antipruritic therapies against this excruciating and frequent symptom not only in hemodialysis patients and for the benefit of patients worldwide is mandatory.

Substance P (SP) is an important mediator in the induction and maintenance of pruritus [11]–[13] and therefore represents an interesting target for antipruritic treatment. SP is a tachykinin which binds with different affinities to three neurokinin receptors (NKR 1–3), but mainly to NKR1, which is expressed in the central nervous system (CNS) and the skin [11]. We therefore investigated in chronic pruritus patients the possible antipruritic potency of the recently developed oral NKR1-antagonist aprepitant.

## Methods

Twenty patients (12 female, 8 male; age range: 36–85 years; mean, 66.7 years; SD +/−13.7; median 68.5 years) with therapy refractory chronic pruritus (duration, 4 months to 20 years; mean, 61.3 months) were randomly selected for the treatment with the selective high-affinity NKR1-antagonist aprepitant. Previous to application of aprepitant, patients underwent clinical investigation to determine the underlying origin of pruritus [3]. All patients had been refractory to at least two (2 to 7) previous antipruritic treatments with topical, intralesional or systemic corticosteroids, antihistamines, and/or UV-irradiation. In 8 patients the origin of chronic pruritus was unclear despite extensive laboratory and radiological investigations (Table 1). In 12 patients underlying diseases were found: chronic kidney disease (uraemic pruritus, n = 5), and a combination of multiple causal factors (chronic kidney disease, diabetes mellitus, hyperuricaemia, and iron deficiency, n = 7). 10/20 patients had additionally an atopic diathesis (four with pruritus of unknown origin and six with pruritus of multifactorial origin). Clinically, 13/20 patients suffered from severe scratch lesions (prurigo nodularis), while seven patients reported chronic pruritus on clinically normal skin. Patients received a monotherapy with aprepitant 80 mg once daily for 3–13 days (mean, 6.6 days) without any other concomitant antipruritic therapy. Patients recorded on a daily base the average pruritus intensity on the visual analog scale (VAS) ranging from 0 (no pruritus) to 10 (severe pruritus). In addition, at the end of treatment, patients were interviewed on the total percentage of change in pruritus (100% reduction  =  complete relief of pruritus).

**Table 1:** Collective of patients: demographic, clinical and response parameters.

No.	Age (years), Gender	Diagnosis*, Origin of pruritus	Atopic diathesis+	Initial pruritus intensity on VAS (ranging 0 (best) to 10 (worst))	Response: reduction of pruritus in percent
1	68, f	CP*, renal	−	6	0
2	82, m	CP, renal	−	10	0
3	69, m	PN*, renal	−	7	30
4	72, m	CP, renal	+	7	40
5	78, m	CP, renal	+	8	50
6	82, f	PN, multifactorial (renal, hyperuricaemia)	−	10	20
7	66, f	PN, multifactorial (renal, dry skin)	+	5	40
8	55, f	CP, multifactorial (cholestatic, dry skin, psychosomatic factors)	+	10	50
9	73, f	PN, multifactorial (renal, diabetes)	+	10	60
10	59, m	PN, multifactorial (metabolic syndrome)	−	10	70
11	42, m	PN, multifactorial (thyreoid dysfunction, neurogenic)	−	8	90
12	66, f	PN, multifactorial (hyperuricaemia, iron deficiency)	+	8	100
13	68, f	PN, unknown	+	10	0
14	77, f	CP, unknown	−	10	0
15	81, f	PN, unknown	−	7	10
16	85, f	PN, unknown	−	10	10
17	36, m	CP, unknown	−	8	40
18	52, f	PN, unknown	+	10	50
19	72, m	PN, unknown	+	6	50
20	50, f	PN, unknown	+	8	100

*CP, chronic pruritus; PN, prurigo nodularis.

+ Atopic diathesis; +, present; −, not present.

Patients' data were anonymously statistically analysed with the SPSS software package (Version 14.0, SPSS Inc., Chicago, Illinois, USA). Paired T-test was applied and p values less than 0.05 were considered statistically significant. Patients were treated individually with aprepitant and gave written informed consent to the off-label application of the drug. The data were encoded in our multidisciplinary pruritus database and retrospective analysis of the study was authorized by the local ethics committee (ethics committee of the medical association of Westphalia and medical faculty of the Westphalian Wilhelms University Münster).

## Results

**Figure 1:**
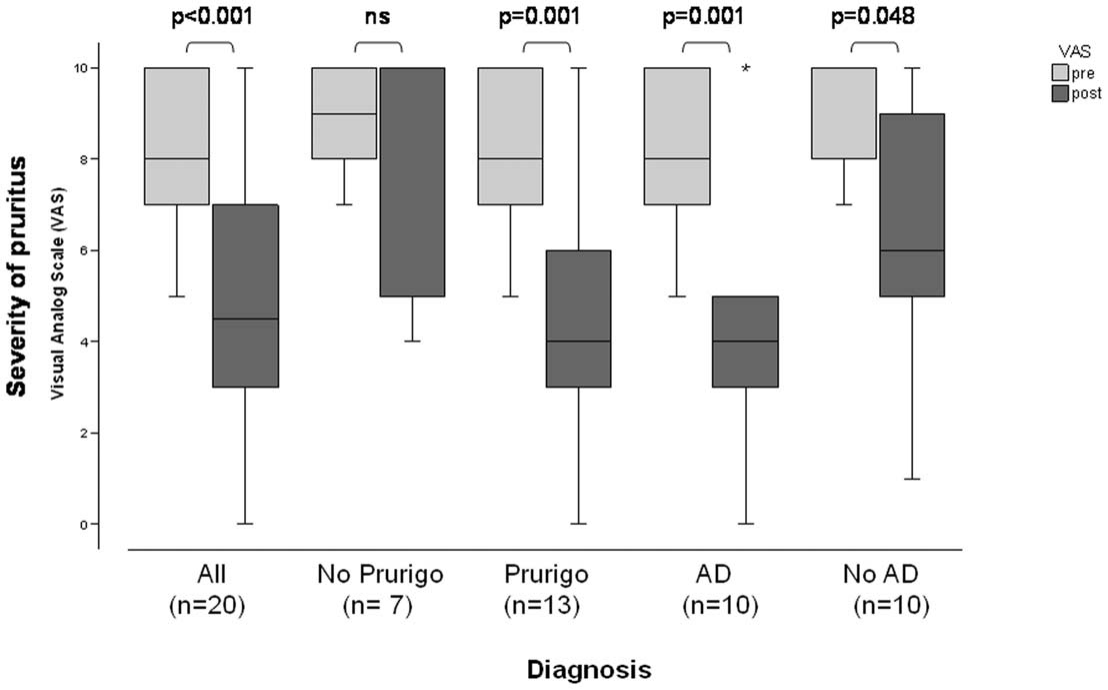
Distribution of values for pruritus intensity as scored on the visual analog scale (VAS) from 0 to 10 before (pre) and after (post) aprepitant. Response is shown for all patients (All, n = 20) as well as for several diagnostic subgroups: patients without (*No Prurigo*) or with (*Prurigo*) clinical presence of chronic scratch lesions as well as patients with (*AD*) and without (*No AD*) atopic predisposition. Best antipruritic effects were observed in patients with atopic predisposition and prurigo nodularis. Bar: median response in each group.

Sixteen out of 20 patients (80%) responded to short-term aprepitant monotherapy. Four patients experienced complete (100% reduction) or nearly complete (70–90%) cessation of pruritus, eight patients reported on partial reduction (40–60%) and four patients experienced weak reduction (10–30%) of pruritus within the treatment period (mean, 50.6% pruritus reduction). Four patients did not respond to the treatment. Pruritus intensity on the VAS before treatment ranged from 5 to 10 (mean, 8.4 points; SD +/−1.7; median VAS 8). After treatment with aprepitant, pruritus intensity was reduced to a mean of 4.9 points (SD +/−3.2; p<0.001; CI 1.913–5.187, Fig. 1). Patients with dermatological diseases appeared to respond best to therapy with aprepitant. Patients with atopic diathesis (n = 10) or prurigo nodularis showed (n = 13) significant reduction of pruritus (p = 0.001; Table 2). The mean reduction of pruritus in atopic patients was 54% (p = 0.001; CI 2.144–6.656, Fig. 1). Patients without atopic diathesis (n = 10) experienced weak reduction of pruritus of 27% (p = 0.048; CI 0.025–5.086, Fig. 1). In the group of patients with prurigo nodularis, mean VAS reduction was 48.5% (p = 0.001; CI 1.863–6.137, Fig. 1) ultimately also leading to clinical improvement of scratch lesions. Patients without scratch lesions showed pruritus reduction of 25.7% (p = 0.094; CI-0.554–5.554, Fig. 1). Patients with systemic origin of pruritus such as chronic kidney disease (uraemic pruritus) responded only weakly to aprepitant (mean reduction, 24.0%). However, patients with uraemic pruritus and additional atopic diathesis showed a much better response, reporting a mean reduction of pruritus of 50%. Moreover, also prurigo nodularis patients responded better than patients without scratch lesions in patient subgroups with the same origin of pruritus (e.g., pruritus of unknown origin).

**Table 2:** Antipruritic effects in patients with or without atopic diathesis.

Atopic diathesis	Pruritus in chronic kidney disease number of patients/patients with response	Multifactorial origin of pruritus number of patients/patients with response	Pruritus of unknown origin number of patients/patients with response	Total Response
Present	2/2	4/4	4/3	9/10
Not present	3/0	3/2	4/1	3/10

Interestingly, patients aged between 36 and 60 years (n = 6) responded significantly better (mean pruritus reduction, 66.7±24.2%; median 60%) than elderly patients aged over 65 years (n = 14; mean pruritus reduction, 29.3±29.5%; median 25%; p = 0.012). Gender analysis did not reveal a significant difference in the response though men tend to respond better (n = 8; mean pruritus reduction, 46.3±26.7%; median 45%) than women (n = 12; mean pruritus reduction, 36.7±36.5%; median 30%; p = 0.507).

In sum, therapy with aprepitant leads to pruritus reduction mainly in dermatological diseases such as atopic diathesis and prurigo nodularis. Side-effects remained mild (nausea, vertigo, and drowsiness) and occurred in three patients. In none of these patients cessation of aprepitant therapy was required.

## Discussion

Our patients with chronic, yet therapy refractory pruritus, experienced a significant, antipruritic effect (p<0.001) upon monotherapy with the NKR1-antagonist aprepitant within one week. This is the first clinical case series demonstrating that targeting the neuropeptide SP via applying the NKR1-antagonist aprepitant is an effective approach for the treatment of chronic pruritus in patients who all had not responded to previous therapies with topical, intralesional or systemic corticosteroids, antihistamines, and/or UV-irradiation. Those patients who had responded were extremely satisfied, gained new hope and confirmed a positive change in their quality of life since they had a long history of vexing pruritus and documentation of several futile therapies. The most significant response rate was observed in patients with atopic diathesis or clinical presentation of chronic scratch lesions of prurigo nodularis. This is further supported by previous findings of increased SP serum levels in patients with atopic dermatitis, which correlated with the pruritus intensity [14]. Moreover, atopic dermatitis and prurigo nodularis were reported to be characterized by increased SP-positive skin nerve fibers [15], [16], which might explain the high response rate in our patients with these disorders. The observation of aprepitant exhibiting a significant antipruritic activity particularly in inflammatory and pruritic skin diseases was confirmed by a recent report. Accordingly, upon treatment with aprepitant a rapid and pronounced improvement was observed in three patients suffering from cutaneous T-cell lymphoma [17]. The results in our patients are promising enough to warrant assessing the efficacy of this novel effect of aprepitant in a randomized, controlled clinical trial.

Most importantly, the antipruritic effect was already observed as early as two days after initiating treatment. This rapid cessation of chronic pruritus in patients with a long history of pruritus (mean, 61.3 months) argues against a placebo effect or spontaneous remission in chronic pruritus patients who had not responded to multiple pre-treatments. The early onset of action suggests that aprepitant acts as a target-specific therapy. Experimental studies clearly showed that SP is involved in pruritus induction in animals and man [12], [18]. Injected into the skin, SP rapidly induced pruritus in both normal and experimentally-evoked inflamed skin in non-atopic healthy volunteers [18]. In mice, SP injections resulted in a dose-dependent increase in scratching of the injected site [12]. The induction of pruritus is due to the SP-induced production of pro-inflammatory cytokines and release of pruritogenic mediators from mast cells such as histamine, tumor necrosis factor (TNF)-α, prostaglandin D2, and leukotriene B4 via binding of SP to NKR1 on keratinocytes and mast cells [13], [19], [20]. The neutralization of these effects by aprepitant most likely suppresses the release of pruritogenic mediators involved in induction and maintenance of chronic pruritus. Moreover, previous experimental studies demonstrated that SP-antagonists may be beneficial substrates for the treatment of inflammatory skin diseases in animal models [21], [22].

Aprepitant is a selective high-affinity NKR1-antagonist with little or no affinity for other neurokinin receptors. It was developed and approved in 2003 for the prevention of chemotherapy-induced emesis and is usually administered for three days only [23], [24]. However, long-term application of the compound for up to six or eight weeks was reported to be safe in a previous studies [25]. Since in long-term studies usually 80 mg were applied, we also decided to use this dosage. Thus, it needs to be investigated whether increasing the dosage and/or the therapeutic interval may increase the antipruritic effect. Aprepitant crosses the blood-brain barrier to mediate antiemetic effects in the CNS, most likely in the chemoreceptor trigger zone in area postrema at the base of the fourth ventricle [23]. Although pruritus can be induced by SP injection into the CNS in animals [26], recent central imaging studies in humans showed that major cerebral areas for pruriception are the somatosensory cortex, midcingulate gyrus, and prefrontal area [27] but not the fourth ventricle. Therefore, it may be speculated that the antipruritic effect of aprepitant is mainly mediated in the skin but not in the CNS. This speculation is underlined by the failure of pruritus relief in systemic diseases such as chronic kidney disease upon application of aprepitant. Due to a potential role of SP in depression and pain including mediation of neurogenic dural inflammation, it was speculated that aprepitant is effective in central and peripheral pain syndromes [28]. However, aprepitant failed to relieve pain in clinical and experimental studies such as the electrical hyperalgesia model [29]. Clinical trials investigating the efficacy of aprepitant in depression did not show significant benefit in safe, non-toxic doses leading to interruption of development and approval of NKR1-antagonists for this indication [26]. Recent studies clearly showed separate pathways for itch and pain [1]. SP plays an important role in both pain and pruritus pathways [1]. Interestingly, animal studies suggest that the neuropeptides SP and calcitonin gene-related peptide (CGRP) play an opposite role in pain and pruritus. While enhanced SP levels have been demonstrated to be related to reduced CGRP levels in an atopic dermatitis mouse model [30], pain studies showed a correlation to CGRP levels [31]. It might therefore be speculated that SP has a differential role in pain and pruritus possibly explaining opposite response in pain (failure of aprepitant) and pruritus (high response to aprepitant). It therefore seems likely that application of an NKR1 antagonist in SP-mediated pruritogenic diseases targets specific pruritus-related neuronal pathways different from pain pathways.

In conclusion our findings outline that aprepitant presents a novel, promising therapeutic approach of pruritus which is especially effective in patients with chronic pruritus due to atopic predisposition/dermatitis or prurigo nodularis. The tendency towards better response in young (male) patients suggest aprepitant to be a novel therapeutic option in adult but not elderly patients with atopic dermatitis.
